# Supplementary material for: The Thioredoxin System in Edwardsiella piscicida Contributes to Oxidative Stress Tolerance, Motility, and Virulence
Source: Microorganisms. 2023 Mar 24;11(4):827. doi: 10.3390/microorganisms11040827 (PMC10145099; doi:10.3390/microorganisms11040827)
Supplement: Supplementary file 1 [file microorganisms-11-00827-s001.zip › microorganisms-2245696-supplementary.pdf]

Supplementary Materials: The Trx system in *Edwardsiella piscicida* contributes to oxidative stress tolerance, motility, and virulence

Jiaojiao He, Su Liu, Qingjian Fang, Hanjie Gu, Yonghua Hu

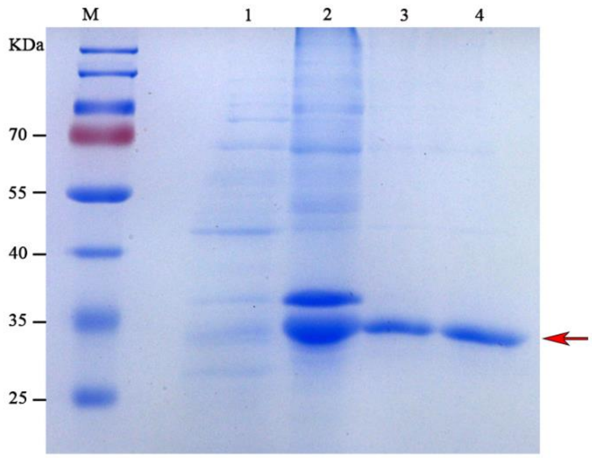

Figure S1. Expression and purification of rTrxB$_{Ep}$. The rTrxB$_{Ep}$ carrying His-Tag is highly purified by affinity chromatography with Ni-NTA. The band indicated by the red arrow is rTrxB$_{Ep}$ protein, which mainly appears in the SDS-PAGE gel stained with Coomassie blue. Lane M: PageRuler Prestained Protein Ladder (Thermo Scientific, US); lane 1: non-induced sample; lane 2: IPTG-induced sample; lane 3: purified sample; lane 4: renatured sample.

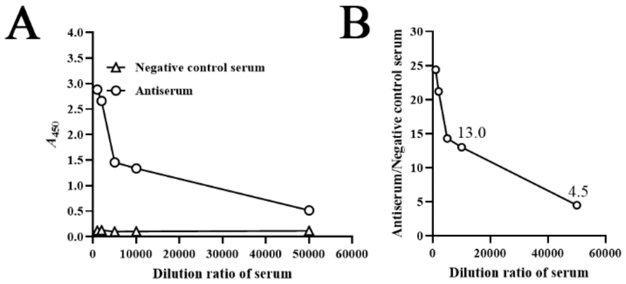

Figure. S2. Determination of antiserum titer by ELISA. **A**, the antibody reacted with purified rTrxB$_{Ep}$ protein by continuous dilution (from 1000-fold, 2000-fold, 5000-fold, 10000-fold to 50000-fold). The unimmunized mice serum was used as a negative control. **B**, the ratio of absorbance between anti-rTrxB$_{Ep}$ serum and negative control serum. When the dilution ratio was 1:50000, the absorbance ratio of anti-rTrxB$_{Ep}$ serum to negative control serum was 4.5.

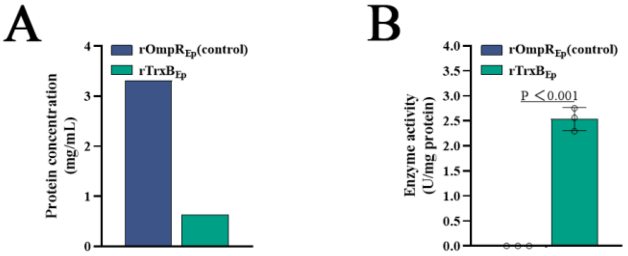

Figure. S3. The thioredoxin reductase activity of the rTrxB$_{Ep}$. **A,** the content of rOmpR$_{Ep}$ (control) and rTrxB$_{Ep}$ was measured using the BCA Protein Assay. **B,** based on OD_{412} analysis the activity of thioredoxin reductase compared with the control rOmpR$_{Ep}$.

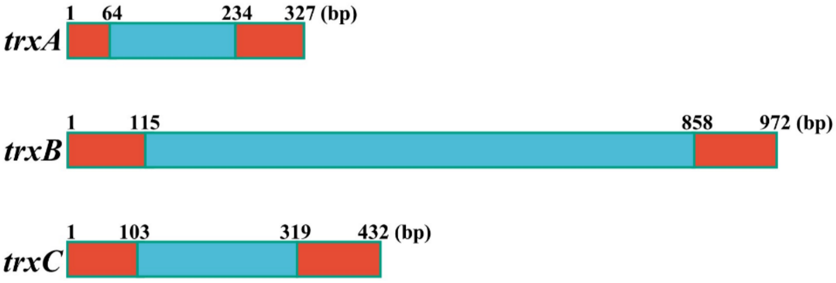

Figure. S4. The sequences knockout location map of *trxA*, *trxB*, *trxC*. Retained sequences are indicated by red shapes and knocked-out regions are indicated by blue shapes.

Table S1 Strains, plasmids used in this study

Strains or plasmids	References or sources
Edwardsiella piscicida	
TX01	Isolated from diseased Japanese flounder
Δ*trxB*	This study
Δ*trxA*	This study
Δ*trxC*	This study
Escherichia coli	
DH5*α*	TransGen Biotech (Beijing, China)
BL21 (DE3)	TransGen Biotech (Beijing, China)
D1000	Biomedal (Sevilla, Spain)
S17-1 *λ*pir	Biomedal (Sevilla, Spain)
BL21/pETTrxB	This study
Plasmids	
pEASY®-T1 Simple Cloning Kit	TransGen Biotech (Beijing, China)

Table S2. Oligonucleotide primers used in this study

Primer name	References or sources Sequence (5′-3′)
TrxBKOF1	GGATCCCATCAGATAAGAGCAAAC (*Bam*H I)
TrxBKOR1	TCCATGACCATGCCGGTGATCAGCAC
TrxBKOF2	CCGGCATGGTCATGGACCATATCTATCGT
TrxBKOR2	GGATCCGACTGCGCTGTTTCAGC (*Bam*H I)
TrxBKOF3	TCGAGTCCAAGACGTACCAC
TrxBKOR3	ATATTACAACGCTGCTCAAGG
TrxBF	GGATCCATGGTCTATGCGGCGCGT (*Bam*H I)
TrxBR	AAGCTTGTGGCGAGCGGCATC (*Hind* III)
TrxAKOF1	GGATCCCGATCAGGATATCGACACC (*Bam*H I)
TrxAKOR1	AGCAGCAGTTCAGCCTTCAGCACGTC
TrxAKOF2	AGGCTGAACTGCTGCTGTTCAAGAAC
TrxAKOR2	GGATCCATCCTGCAGGATCTCCAG (*Bam*H I)
TrxAKOF3	GTCGGCAAACTTCTGTTCAGTC
